# XRCC2 driven homologous recombination subtypes and therapeutic targeting in lung adenocarcinoma metastasis

**DOI:** 10.1038/s41698-024-00658-y

**Published:** 2024-08-01

**Authors:** Han Gong, Peihe Zhang, Qiang Liu, Yuxuan Tian, Fuxin Chen, Siyi Qian, Chaofeng Tu, Yueqiu Tan, Xingming Hu, Bin Zhang

**Affiliations:** 1 The 1st Department of Thoracic Surgery of Hunan Cancer Hospital & the Affiliated Cancer Hospital of Xiangya School of Medicine, Central South University, Changsha, 4100013 China; 2 Molecular Biology Research Center and Center for Medical Genetics, School of Life Sciences, Central South University, Changsha, Hunan China; 3 Department of Anesthesiology, The Third Xiangya Hospital, Central South University, Changsha, China; 4 Hunan Cancer Hospital and the Affiliated Cancer Hospital of Xiangya School of Medicine, Central South University, Changsha, Hunan China; 5 Institute of Reproductive and Stem Cell Engineering, NHC Key Laboratory of Human Stem Cell and Reproductive Engineering, School of Basic Medical Sciences, Central South University, Changsha, Hunan China; 6 Department of Histology and Embryology, School of Basic Medical Sciences, Central South University, Changsha, China; 7 Clinical Research Center for Reproduction and Genetics in Hunan Province, Reproductive and Genetic Hospital of CITIC-Xiangya, Changsha, Hunan China; 8 College of Life Sciences, Hunan Normal University, Changsha, China

**Keywords:** Non-small-cell lung cancer, Cancer

## Abstract

Lung adenocarcinoma (LUAD) is a leading cause of cancer mortality, with many patients facing poor prognosis, particularly those with metastatic or drug-resistant tumors. Homologous recombination genes (HRGs) are crucial in tumor progression and therapy resistance, but their clinical significance in LUAD is not well understood. In this study, we systematically characterize key HRGs in LUAD patients, identifying two distinct HR subtypes associated with different outcomes and biological functions. We establish a 5-gene scoring system (XRCC2, RAD51, BRCA1, FANCA, and CHEK1) that reliably predicts patient outcomes and immunotherapy responses in LUAD. Bioinformatics analysis and clinical validation highlight XRCC2 as a crucial biomarker in LUAD. Functional investigations through in vivo and in vitro experiments reveal the role of XRCC2 in promoting lung cancer migration and invasion. Mechanistically, XRCC2 stabilizes vimentin (VIM) protein expression through deubiquitylation. We predict c-MYC as a potential regulator of XRCC2 and demonstrate that inhibiting c-MYC with compound 10058-F4 reduces XRCC2 and VIM expression. Preclinical studies show the synergistic inhibition of metastasis in vivo when combining 10058-F4 with doxorubicin (Dox). Our findings present a potential personalized predictive tool for LUAD prognosis, identifying XRCC2 as a critical biomarker. The c-Myc-XRCC2-VIM axis emerges as a promising therapeutic target for overcoming lung metastasis. This study provides valuable insights into LUAD, proposing a prognostic tool for further clinical validation and unveiling a potential therapeutic strategy for combating lung metastasis by targeting c-Myc-XRCC2-VIM.

## Introduction

Lung adenocarcinoma (LUAD) is one of the most prevalent and lethal types of lung cancer. While treatment strategies have improved outcomes, the prognosis remains poor for many patients, especially those with metastatic or chemotherapy-resistant tumors^1^. This heterogeneous nature of LUAD poses challenges for management and underscores the need to better stratify patients.

Genomic instability, arising from defects in DNA damage repair, is a hallmark of cancer. Homologous recombination (HR) plays a vital role in maintaining genomic integrity by facilitating error-free repair of DNA double-strand breaks^2^. This process is mediated by key homologous recombination genes (HRGs) such as RAD51, BRCA1/2, and members of the XRCC family^3–5^. Dysregulation of HRGs can compromise repair mechanisms and promote tumorigenesis through increased genomic instability^6,7^. Indeed, mutations in RAD51/BRCA1/2 confer increased cancer risk and impact chemosensitivity in several cancer types^8–10^. The XRCC family was significantly upregulated in non-small cell lung cancer and hepatocellular carcinoma^11,12^. While HRGs are recognized as important factors in cancer, their clinical relevance and underlying mechanisms in cancer, especially LUAD, remain poorly understood.

Furthermore, HRGs alterations are associated with anti-tumor drug susceptibility^13,14^. A famous example is that patients with BRCA1 and BRCA2 mutations are more sensitive to poly (ADP-ribose) polymerase (PARP) protein inhibitors^6^. Additionally, overexpression of XRCC2 reduced cell cycle arrest and enhanced the sensitivity to chemoradiotherapy in lung cancer cells^15^. Current chemotherapy with doxorubicin (Dox) is limited by toxicities and emerging resistance^16,17^. As HRG alterations can affect drug response, understanding their roles in LUAD may help address these challenges.

In this study, we aimed to address gaps in understanding how HRGs clinically impact LUAD, with the goal of elucidating novel stratification tools and therapeutic vulnerabilities for overcoming the lethality of this disease. We investigate the expression and prognostic values of HRGs in LUAD patients and define distinct HR subtypes associated with outcomes. Additionally, we sought to develop a prognostic HR scoring system and identify key biomarkers and targets mediating LUAD pathogenesis. Based on bioinformatics findings, we focused on characterizing the functions of XRCC2 in LUAD. Through in vitro and in vivo experiments, we explored the hypothesis that XRCC2 promotes metastasis and its mechanisms of action. Finally, we examined a potential treatment approach targeting the c-Myc-XRCC2-VIM axis.

## Results

### Characterization of 18 key HRGs in LUAD

**Fig. 1:**
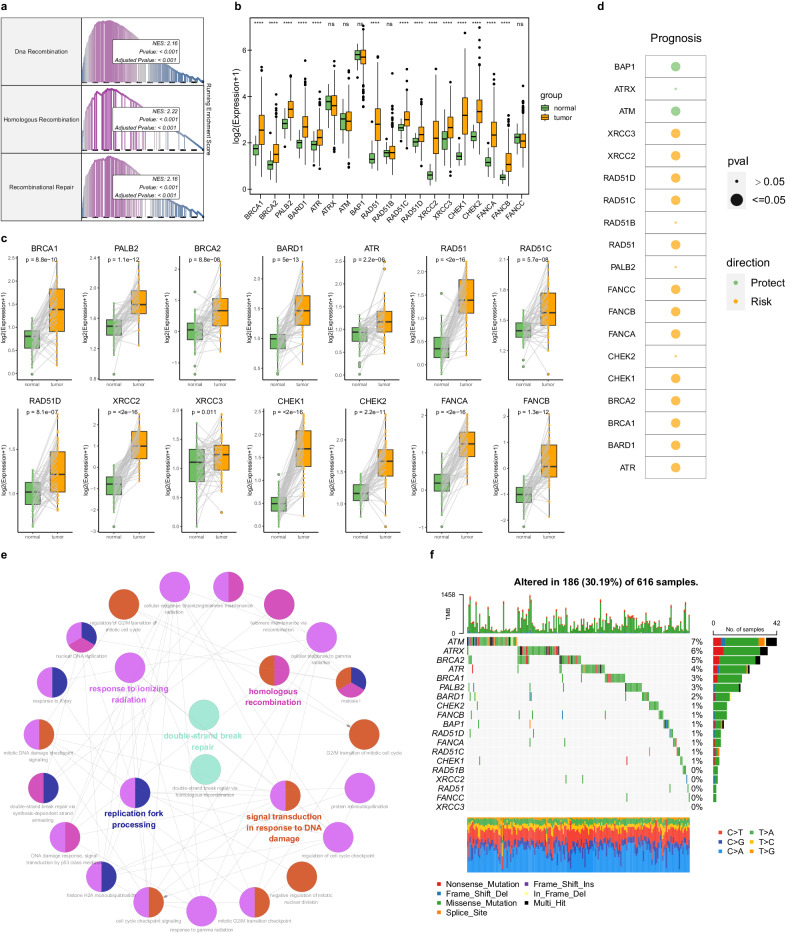
Comprehensive analysis of 18 key HRGs in LUAD. **a** The GSEA algorithm showed that the DNA recombination, homologous recombination, and recombinational repair pathways were significantly enriched in LUAD. **b** Comparative analysis of expression levels for the 18 HRGs between tumor and normal tissues. **c** Elevated expression of 14 HRGs in paired samples. **d** Prognostic analysis of 18 HRGs using the log-rank test for survival curves. **e** Functional enrichment network of 18 HRGs elucidated through the ClueGO algorithm. **f** Exploration of mutation frequency and types for each HRG in LUAD patients. The boxplots show the distribution of expression levels for distinct groups. The center line represents the median. The box boundaries represent the interquartile range. The whiskers representing the minimum and maximum values.

To quantify differences in HR pathway activity between tumor and normal samples in LUAD, we conducted GSEA to analyze the enrichment of three predefined HR-related terms. The results showed that HR pathways were significantly enriched in LUAD (Fig. 1a), which highlighted their potential biological and therapeutic relevance. The boxplot comparison of 19 key HRGs revealed 14 genes with significantly higher expression in tumors (Fig. 1b). Further analysis of paired samples confirmed the upregulation of these 14 HRGs in cancer relative to adjacent normal lung tissue (Fig. 1c). To explore the prognostic relevance of the HRGs, we performed the OS analysis to evaluate the indicators of the HRGs in LUAD patients. The results suggested that 13 HRGs were risk factors while 2 HRGs were protective factors (Fig. 1d). The functional annotation network confirms that these HRGs were mainly enriched in HR related pathways (Fig. 1e). Additionally, we assessed the mutation frequencies of HRGs in LUAD. The corresponding waterfall plot revealed that the three most frequently mutated genes were ATM, ATRX, and BRCA2 (Fig. 1f).

### Identification of two HR-related subtypes in LUAD

**Fig. 2:**
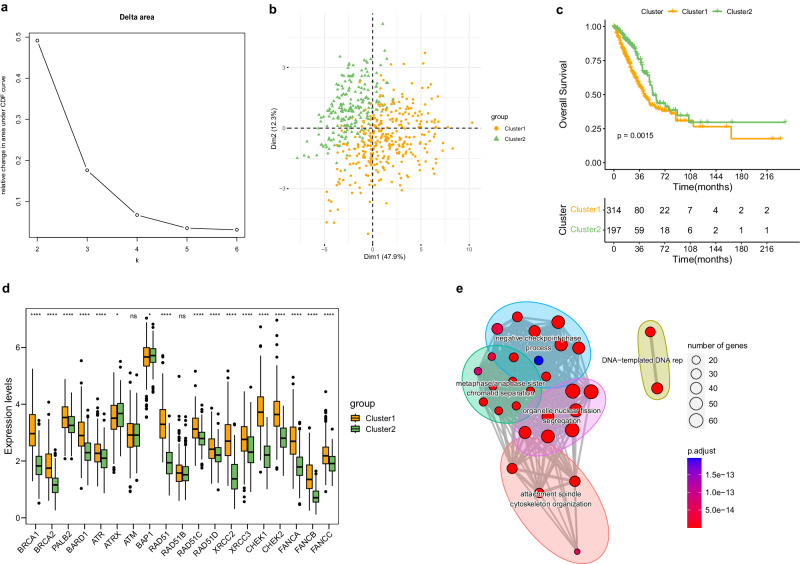
Uncovering two prognostic HRG-defined subgroups in LUAD. **a** Evaluating cluster numbers from k = 2 to 6 identified two as the optimal clustering via CDF curve analysis. **b** PCA plot visualized clear separation of the Cluster 1 and Cluster 2 based on 18 HRG expression profiles. **c** KM curves conducted to compare outcomes between the Cluster 1 and Cluster 2. **d** Comparison of 18 CFRGs expression between the two identified subtypes. **e** Functional grouping network of GO term enriched by DEGs. *p < 0.05, ****p < 0.0001. The boxplots show the distribution of expression levels for distinct groups. The center line represents the median. The box boundaries represent the interquartile range. The whiskers representing the minimum and maximum values.

In clinical practice, an effective approach to optimize and enhance clinical care involves categorizing patients based on their HR status^18^. To investigate the heterogeneity associated with HR in patients with LUAD, we employed the consensus clustering algorithm to identify distinct subtypes. The cumulative distribution function (CDF) curve analysis demonstrated that the transition from K = 2 to K = 3 yielded the greatest relative change in the area under the CDF curve (Fig. 2a), indicating that the optimal HR-related subtype corresponds to K = 2. Supporting this, the PCA plot visually confirmed the distinct distributions between the two HR-related LUAD subtypes (Fig. 2b). Furthermore, the analysis of OS revealed significantly lower survival probabilities for Cluster 1 compared to Cluster 2 (Fig. 2c). The two HR subtypes exhibited different expression patterns of the HRGs (Fig. 2d). Moreover, enrichment analysis highlighted the significant enrichment of pathways associated with DNA replication and chromosome segregation (Fig. 2e). These findings contribute to a better understanding of HR-related heterogeneity in LUAD patients and have implications for personalized clinical management and treatment strategies.

### Development of a HR scoring system and application in pan-cancer

**Fig. 3:**
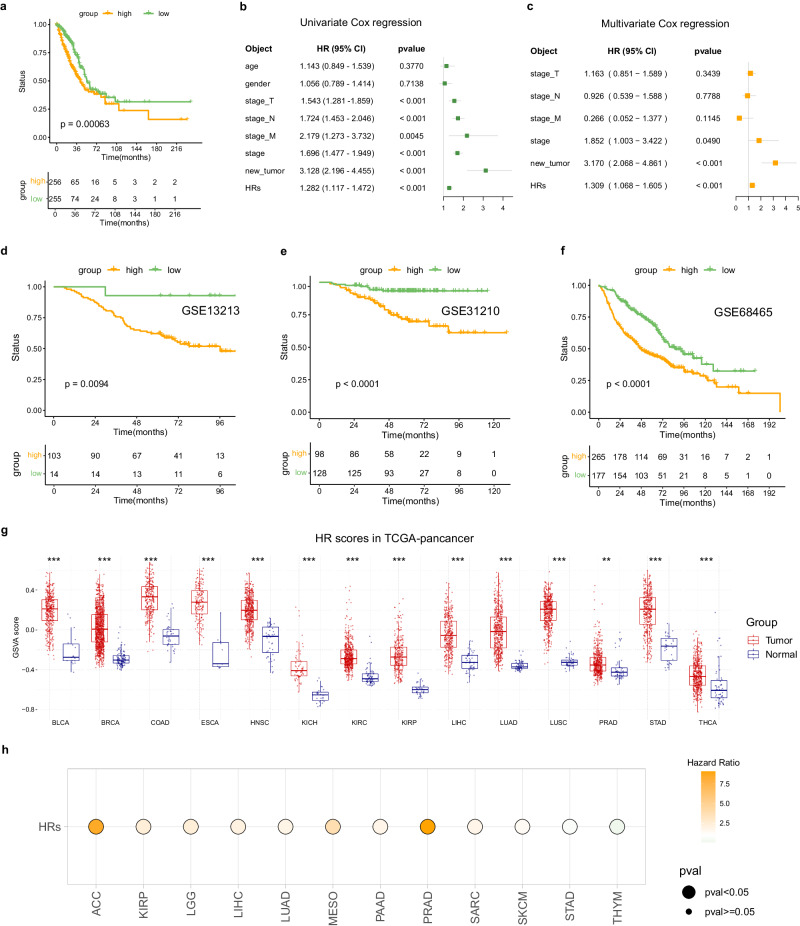
Construction and pan-cancer application of the HR scoring system. **a** KM curves showed significantly poorer outcome for LUAD patients with high HR scores (*p* < 0.001, log-rank test). **b**, **c** Univariate and multivariate Cox analyses identified the HR score as an independent prognostic biomarker. **d**–**f** External validation on independent cohorts. **g** A pan-cancer comparison revealed an elevated HR scores in diverse tumors versus normal tissues. **h** The hazard ratio of the HR scoring system in pan-cancer, selectively displaying tumor categories with significant prognostic value in HR scores. **p < 0.01, ***p < 0.001. The boxplots show the distribution of expression levels for distinct groups. The center line represents the median. The box boundaries represent the interquartile range. The whiskers representing the minimum and maximum values.

To personalize the assessment of HR impact on patient prognosis, we developed a HR scoring system utilizing the GSVA algorithm. KM curves displayed a significant association between high HR scores and unfavorable prognosis (Fig. 3a). Furthermore, combined univariate and multivariate Cox analyses demonstrated that the HR scores served as an independent prognostic factor, irrespective of other clinicopathological features, thereby showcasing its clinical applicability (Fig. 3b, c). To assess the robustness of the HR scoring system, we applied it to three independent LUAD cohorts. KM curves were generated, revealing that the high HR subgroup exhibited poorer outcomes in the validation set (Fig. 3d–f).

Pan-cancer analysis helps overcome the inherent restrictions of studying individual cancer types in isolation, leading to insights that can have implications for multiple cancer types and improve patient care on a larger scale^19^. To better understand the role of HR scores in pan-cancer, we conducted a systematic analysis of HR scores between tumor tissues and normal tissues using the GSCA database. Intriguingly, we observed a consistent upregulation of HR scores across all cancer types (Fig. 3g). Additionally, our further analysis of OS revealed a strong association between high HR scores and poor prognosis in 11 different types of cancer, while only showing a favorable prognosis in THYM (Fig. 3h). These findings highlighted that high HR scores served as a risk factor in LUAD and had potential implications in various other malignancies. Our results suggest that the HR scoring system hold promise as a pan-cancer prognostic model with wide-ranging clinical applicability.

### Comprehensive comparison of the two subgroups in biological function, genomic alteration, and immunotherapy effect

**Fig. 4:**
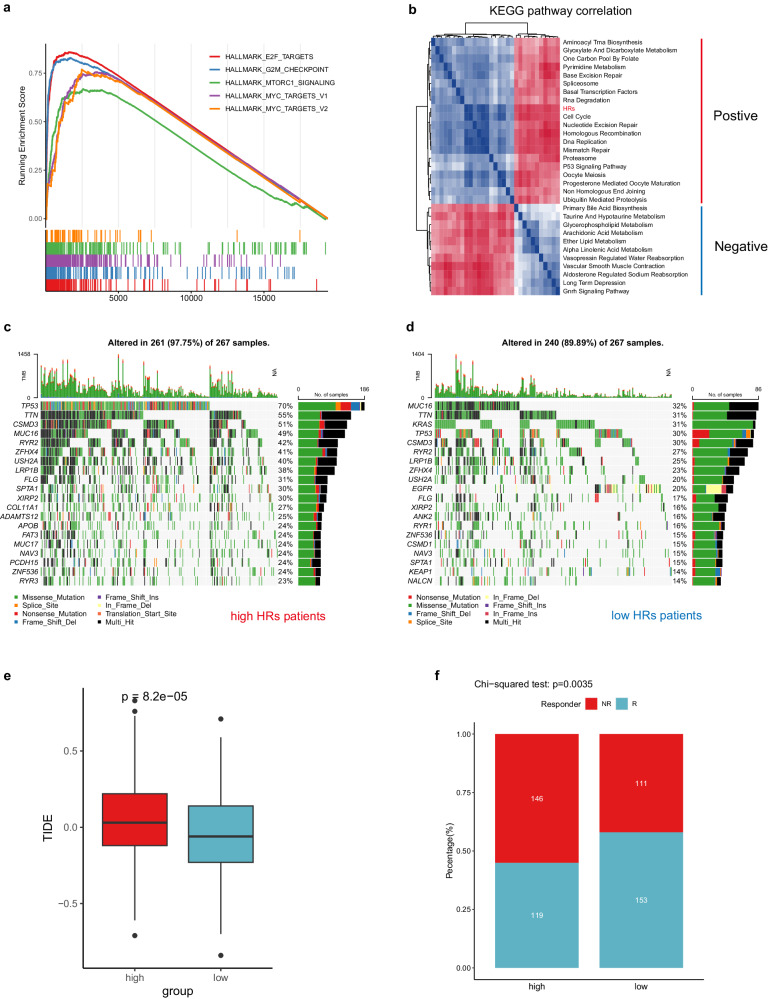
Comprehensive comparison of the two categories of LUAD patients defined by HR score. **a** Identification of top 5 hallmark pathways associated with HR scores using the GSEA method. **b** Heatmap displaying KEGG terms with an absolute correlation value with HR score greater than 0.5. **c**, **d** Mutation profiles uncovered differences in mutation burden between subgroups. **e** Comparative analysis of TIDE score between the two HRs subgroups. **f** Stacked bar plot indicating a more favorable immunotherapy efficacy in the low-HR subgroup. The boxplots show the distribution of expression levels for distinct groups. The center line represents the median. The box boundaries represent the interquartile range. The whiskers representing the minimum and maximum values.

To gain further insights into the molecular characteristics associated with the HR scores in LUAD patients, we conducted GSEA algorithm. Our analysis revealed the top 5 enriched terms in LUAD patients with high HR scores, which included E2F target, G2M checkpoint, MTORC1 signaling, MYC target V1, and MYC target V2 (Fig. 4a). Additionally, we performed Spearman correlation analysis, which demonstrated a positive correlation between the HR score and pathways related to the cell cycle, P53 signaling, DNA replication, and other pathways (Fig. 4b). Conversely, we observed a negative correlation between the HR score and pathways associated with ether lipid metabolism, alpha linolenic acid metabolism, and other pathways (Fig. 4b). We conducted an analysis of single nucleotide variants (SNVs) and observed that the high HR score group exhibited a higher frequencies of gene mutations, particularly in the TP53 gene (high: 70% vs low: 30%) (Fig. 4c, d). Furthermore, we utilized the tumor immune dysfunction and exclusion (TIDE) algorithm to predict the response to immunotherapy in the two subgroups. The results reveal that the high HR score group have higher TIDE scores and a lower response rate to immunotherapy compared to the low HR score group (Fig. 4e, f).

### Validation of XRCC2 by clinical sample

**Fig. 5:**
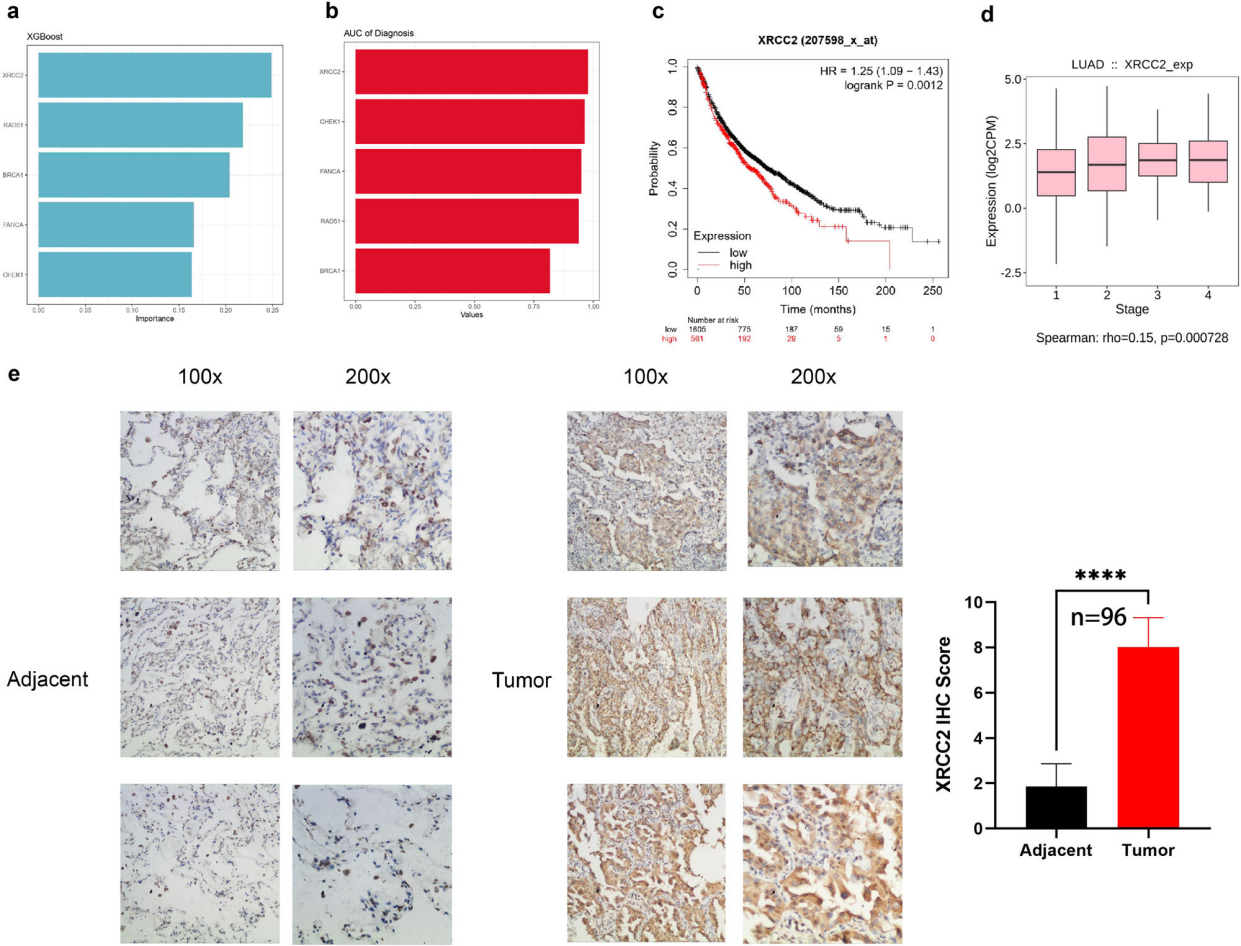
XRCC2 as a potential biomarker in LUAD. **a** The XGBoost algorithm prioritized XRCC2 as the top-ranking prognostic marker. **b** The diagnostic ROC curve highlighted XRCC2 with the highest AUC value. **c** Analysis of an external dataset revealed a significant reduction in prognosis for patients with elevated XRCC2 expression. **d** XRCC2 exhibited elevated expression in advanced stages of LUAD. **e** ICH analysis of clinical samples confirmed the upregulation of XRCC2 (*n* = 96). ****p < 0.0001. The boxplots show the distribution of expression levels for distinct groups. The center line represents the median. The box boundaries represent the interquartile range. The whiskers representing the minimum and maximum values. The error bars represent the standard error of the mean (SEM).

We utilized the XGBoost algorithm to assess the prognostic importance of five HRGs - XRCC2, RAD51, BRCA1, FANCA, and CHEK1. Remarkably, XRCC2 was ranked as the most significant HRG (Fig. 5a). Furthermore, the diagnostic receiver operating characteristic (ROC) curve analysis demonstrated that XRCC2 exhibited the highest efficiency among the five HRGs (Fig. 5b). Additionally, we observed a gradual increase in XRCC2 expression levels with the progression of LUAD (Fig. 5c). Survival analysis conducted on a large cohort of 2166 LUAD patients by KM plotter database^20^ further confirmed that high XRCC2 expression was associated with shorter survival (Fig. 5d), suggesting its potential prognostic value. To further validate the clinical relevance of XRCC2, we collected and analyzed samples from 96 LUAD patients. The immunohistochemistry results revealed significantly higher XRCC2 expression levels in cancer tissues compared to adjacent normal tissues (Fig. 5e), corroborating its role as a tumor-associated biomarker in LUAD. These findings underscore the potential of XRCC2 as a valuable biomarker in LUAD.

### XRCC2 stabilizes VIM expression via deubiquitylation

**Fig. 6:**
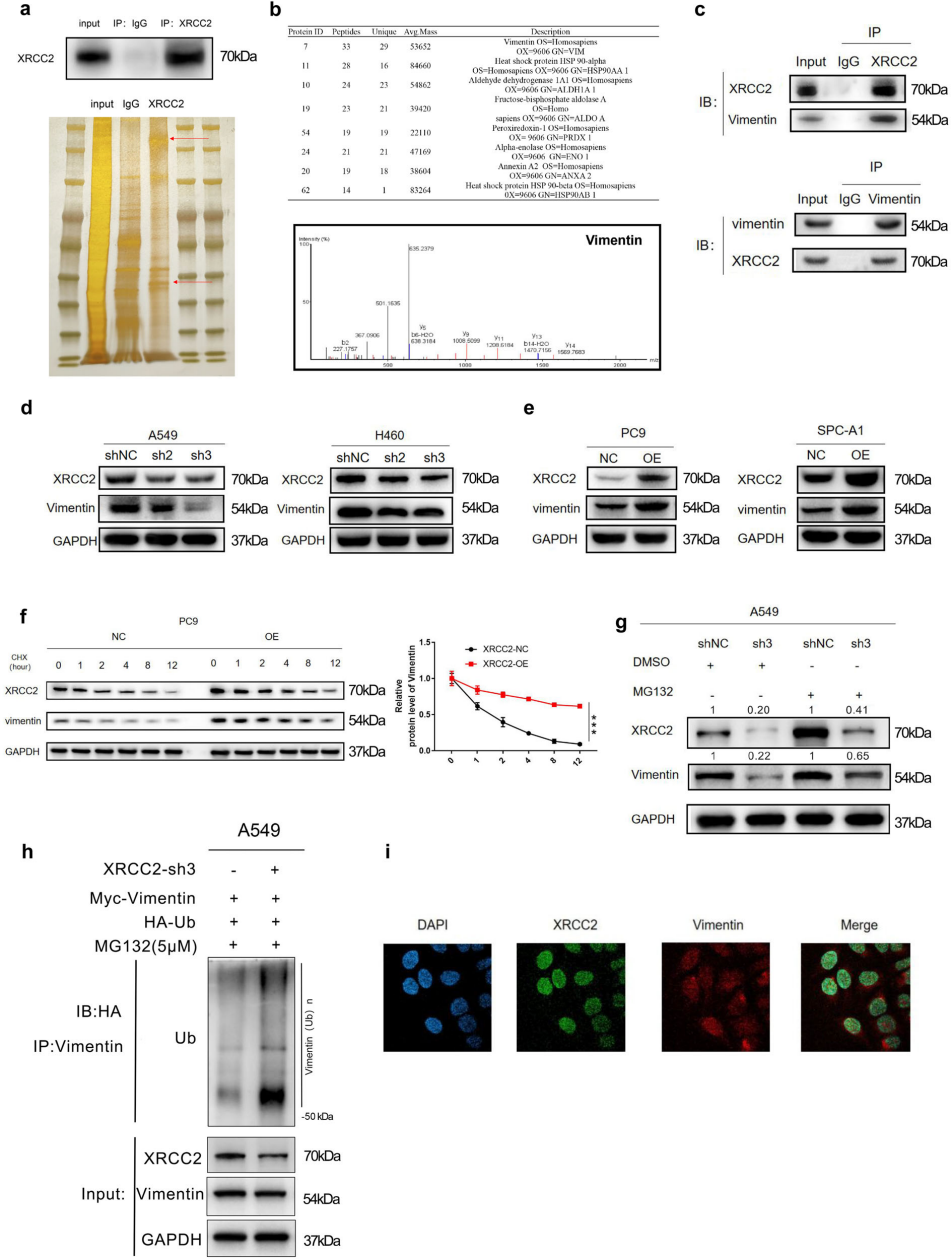
VIM was a downstream interacting protein target of XRCC2. **a** IP experiment capturing XRCC2 interacting proteins for subsequent silver nitrate staining. **b** Scoring and sorting of the main interacting proteins of XRCC2, including the secondary mass spectrum of characteristic peptides of the interacting protein Vimentin. **c** Verification of XRCC2 and vimentin interaction through co-IP assays. **d**, **e** Protein level detection of VIM after XRCC2 knockdown or overexpression, as assessed by Western blot experiments. **f** Following XRCC2 overexpression in PC9 cells, treatment with cycloheximide (CHX) (100 ng/mL) over various time points (0, 1 h, 2 h, 4 h, 8 h, and 12 h) with Western blot used to detect Vimentin expression. NC Negative Control, OE Overexpression XRCC2. **g** XRCC2 knockdown in A549 cells treated with MG132 (10 μM) for 6 h, with Western blot revealing the impact of XRCC2 knockdown on vimentin expression. **h** Western blot analysis of the ubiquitination level of Vimentin protein after XRCC2 knockdown in A549 cells. **i** Co-localization observation of XRCC2 and Vimentin in cells using laser confocal microscopy.

To further investigate the molecular mechanism of XRCC2, we attempted to identify potential binding proteins of XRCC2. Employing tandem affinity purification and LC-MS/MS analysis, we identified vimentin (VIM) as a potential interacting protein of XRCC2 (Fig. 6a, b). To validate this interaction, we conducted co-immunoprecipitation (Co-IP) assays using A549 cell lysates, which confirmed the mutual binding of XRCC2 and VIM (Fig. 6c). Furthermore, knocking down XRCC2 or overexpressing XRCC2 led to downregulation or upregulation of VIM expression (Fig. 6d, e). Cycloheximide (CHX) half-life experiments demonstrated that XRCC2 overexpression extended VIM’s half-life, enhancing its protein stability (Fig. 6f). Further investigations with the proteasome inhibitor MG132 confirmed that XRCC2-mediated regulation of VIM stability operated through the proteasome pathway (Fig. 6e). XRCC2 knockdown in A549 cells significantly increased the ubiquitination level of VIM, substantiating XRCC2’s influence on VIM expression through the ubiquitin-proteasome degradation pathway (Fig. 6h). Confocal microscopy revealed co-localization of XRCC2 (green) and VIM (red) within cells (Fig. 6i). These findings collectively identify VIM as a downstream interacting protein target of XRCC2, with XRCC2 regulating its expression via the ubiquitin-proteasome pathway.

### XRCC2 promotes migration and invasion of lung cancer cells in vitro and in vivo

**Fig. 7:**
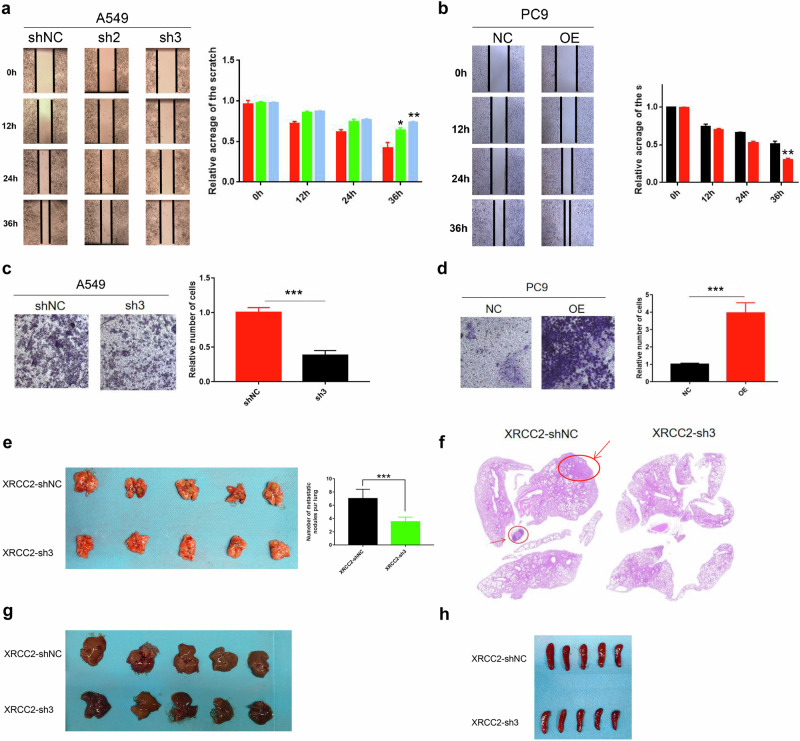
XRCC2 promoted the migration and invasion of lung cancer cells. **a** Scratch experiments after XRCC2-shRNA transfection in A549 cells, recording cell wound healing at 0, 12 h, 24 h, and 36 h. **b** Wound healing assessment in PC9 cells transfected with XRCC2 overexpression plasmid, observed at 0, 12 h, 24 h, and 36 h in scratch experiments. **c** Transwell invasion assay assessing invasion ability of A549 cells, with crystal violet staining and counting after 24 h of chamber culture. **d** Transwell invasion assay evaluating invasion ability of PC9 cells, with crystal violet staining and counting after 24 h of chamber culture. **e** Comparison of metastatic nodules in the lung tissue of nude mice between XRCC2 knockdown and control groups. **f** HE staining to determine the number of lung metastasis nodules in the two groups of nude mice. **g**, **h** Removal of liver and spleen tissues from nude mice, recording and comparing the number of tumor metastasis nodules. **p < 0.01, ***p < 0.001. The error bars represent the SEM.

Vimentin, as an essential cytoskeletal protein, plays a crucial role in epithelial-mesenchymal transition (EMT), a process in which tumor cells acquire migratory and invasive properties, leading to metastasis and chemoresistance^21,22^. Considering the regulatory effect of XRCC2 on vimentin stability, we aimed to investigate the involvement of XRCC2 in lung cancer metastasis. we performed cell scratches and transwell assays in multiple types of lung cancer cells. Knockdown of XRCC2 resulted in a significant decrease while overexpression of XRCC2 resulted in a significant increase in cell migration compared with controls (Fig. 7a, b). At the same time, A similar trend was confirmed in cell invasion experiments (Fig. 7c, d). In vivo experiments, we investigated the impact of XRCC2 on lung metastasis using a mouse model. The results demonstrated that the XRCC2 knockout group exhibited significantly fewer lung metastasis nodules compared to the control group (Fig. 7e, f). Additionally, no metastasis was observed in the liver and spleen of the nude mice in both groups (Fig. 7g, h). These findings provide strong evidence that XRCC2 plays a crucial role in promoting lung metastasis.

### c-Myc as a potential transcription factor for XRCC2

**Fig. 8:**
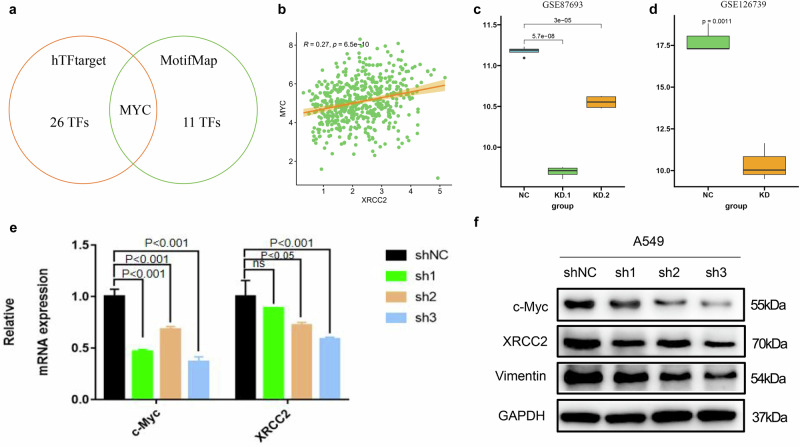
c-Myc served as a potential transcription factor for XRCC2. **a** Analysis of candidate transcription factors for XRCC2 from two transcription factor prediction databases. **b** Spearman correlation analysis revealing a significant positive correlation between XRCC2 and c-Myc. **c**, **d** In public datasets where c-Myc was knocked down, XRCC2 expression was significantly downregulated. **e**, **f** qRT-PCR and Western blot assays conducted to detect XRCC2 expression levels after c-Myc knockdown at both mRNA and protein levels. The error bars represent the SEM.

To identify the regulators upstream of XRCC2, we used bioinformatics tools for TF prediction. We identified 27 potential TFs in the lungs from the hTFtarget database^23^ and 12 potential TFs from the MotifMap database^24^. Combining these two databases, we found that c-Myc, a well-known oncogene, was the only candidate TF (Fig. 8a). Correlation analysis demonstrated that the expression of c-Myc was significantly positively correlated with the expression of XRCC2 (R = 0.27, *p* < 0.001) (Fig. 8b). Additionally, XRCC2 expression was notably downregulated in two MYC knockdown public datasets (GSE87693, GSE126739) (Fig. 8c, d). We then transfected c-Myc knockdown plasmids into A549 cells and assessed XRCC2 mRNA and protein expression. C-Myc knockdown significantly inhibited XRCC2 at both the mRNA and protein levels (Fig. 8e, f). These results suggest c-Myc may directly regulate XRCC2 transcription in lung cancer.

### Targeting the c-Myc-XRCC2-VIM Axis for Therapy

Doxorubicin causes cell death by inducing DNA strand breaks. Research indicates that cells deficient in homologous recombination show heightened sensitivity to Doxorubicin^25^. Based on the clinical relevance of the c-Myc-XRCC2-VIM axis and its association with HR activity, we propose that targeting this axis could be a promising therapeutic strategy for overcoming Doxorubicin resistance in LUAD. To investigate this, we conducted mouse model experiments to evaluate the effects of Doxorubicin alone and in combination with the c-Myc inhibitor 10058-F4 on lung metastasis.

**Fig. 9:**
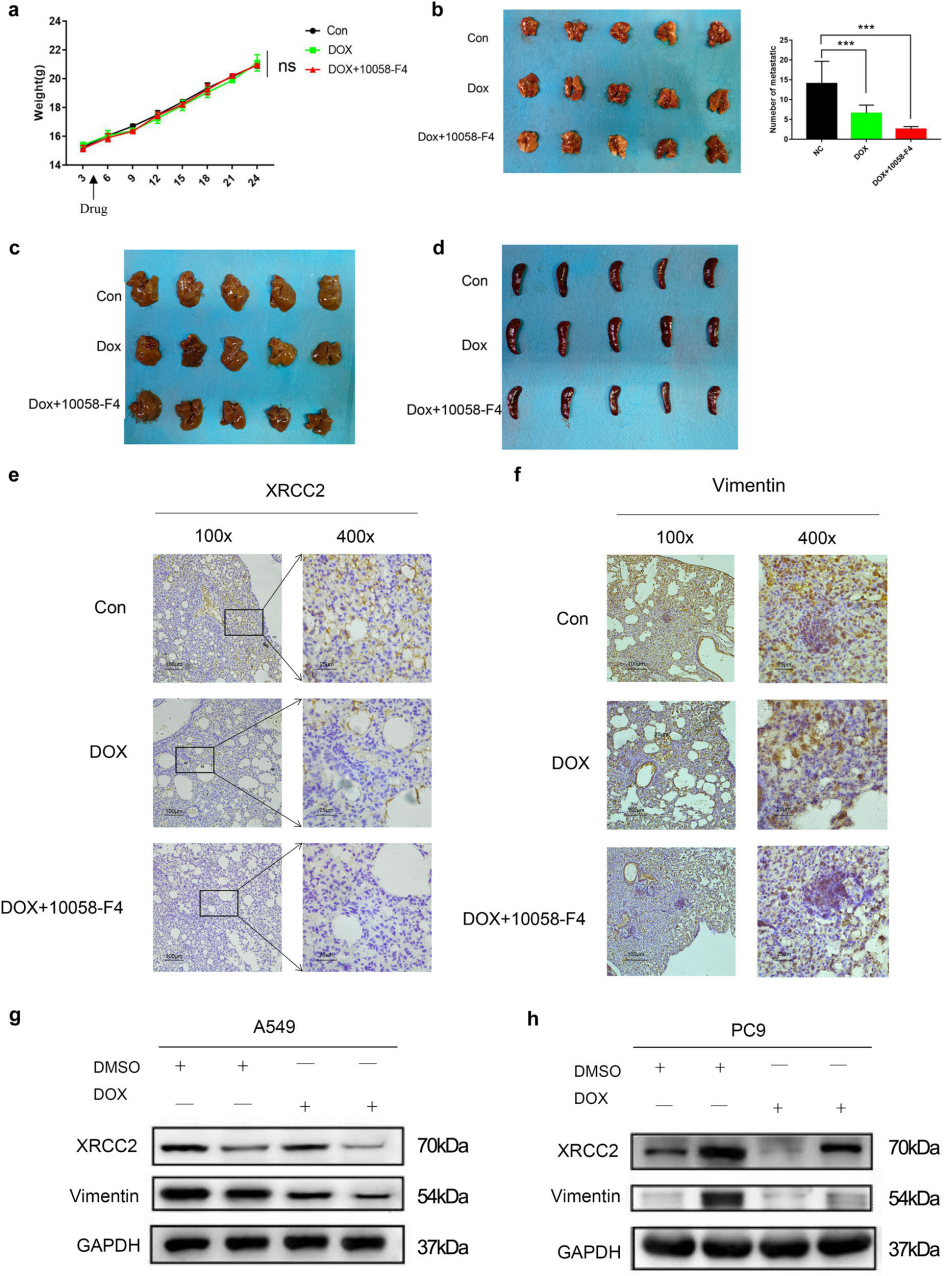
Improved treatment effect with c-Myc inhibitor combined with Doxorubicin (DOX). **a** Measurement and recording of growth curves in two groups of nude mice, illustrating changes in body weight between the control and experimental groups. **b** Lung metastasis comparison between the combined drug group, control group, and single drug group, observed after peeling off the lungs. **c**, **d** Observation of tumor nodules and metastases in the liver and spleen after peeling off these organs. **e**, **f** IHC analysis detecting changes in XRCC2 and Vimentin protein expression in the combined drug group compared to the control and single drug groups. **g** Evaluation of VIM expression after XRCC2 knockdown combined with Dox in A549 cells. **h** Assessment of VIM expression after XRCC2 overexpression combined with Dox in PC9 cells. ***p < 0.001. The error bars represent the SEM.

In Fig. 9a, we observed no significant difference in mouse weight between the experimental group (combination treatment) and the control group, indicating that the treatment did not cause significant toxicity or adverse effects on overall health. When comparing the number of pulmonary metastasis nodules, the combined group exhibited significantly fewer nodules compared to the control group and the Doxorubicin-only group (Fig. 9b). Furthermore, no metastasis was observed in the liver and spleen of the nude mice in both groups (Fig. 9c, d). Immunohistochemical (IHC) analysis indicated that the combination treatment effectively downregulated the expression of XRCC2 and its downstream target VIM (Fig. 9e, f). To further support this, Western blot experiments in A549 and PC9 cells showed that in the XRCC2 knockdown experimental group treated with Doxorubicin, the expression of VIM was further downregulated (Fig. 9g). However, in PC9 cells that overexpressed XRCC2 treated with Doxorubicin, the expression of VIM did not rise to the level of the group that only overexpressed XRCC2 (Fig. 9h). These findings support the hypothesis that targeting the c-Myc-XRCC2-VIM axis could be a promising therapeutic in lung cancer.

## Discussion

This study comprehensively explored the clinical relevance and molecular mechanisms of HRGs in LUAD, supporting their role in tumor progression and outcomes. We developed the novel HR scoring system for LUAD based on five genes, including XRCC2, that were significantly associated with patient prognosis and immunotherapy responses. Tailoring treatments based on the individual HR status of patients aligns with the goals of personalized medicine, offering a more nuanced and effective approach to managing LUAD.

While high HR scores are generally associated with poor prognosis in most tumors, some exceptions exist. In both STAD and THYM, high HR scores were significantly correlated with better prognostic outcomes. Considerable heterogeneity exists between and within cancers^26^. Certain genes may act as tumor suppressors in one cancer but promote tumors in another, such as c-FOS^27^, STAT3^28^, and DDX5^29^. In gastric cancer, Zhang et al. found that BRCA1 loss was linked to higher tumor grade and shorter survival^30^. Additionally, negative nuclear RAD51 staining correlated with lymph node metastasis, larger tumor size, and lower disease-free survival in gastric adenocarcinoma^31^. These findings indicate that HR genes may serve divergent roles across tumor types. Further research is warranted to better understand this phenomenon.

Our identification of XRCC2 as a novel prognostic biomarker and driver of migration and invasion in LUAD provides important evidence for future clinical testing. Prior research had revealed correlation of XRCC2 polymorphisms/expression with tumor risk and drug sensitivity^15,32–35^. Interestingly, a previous proteomic analysis by Rajesh et al. revealed that vimentin is one of the interacting partners of XRCC2^36^. In that study, Rajesh et al. purified the mouse RAD51C, RAD51D and XRCC2 proteins and used them as baits to pull-down interacting partners from mouse embryonic fibroblast cell extracts, which were then identified by mass spectrometry. Our findings align with and extend this knowledge by underlining the role of XRCC2 in promoting metastasis. We mechanistically demonstrated that XRCC2 stabilizes the cytoskeletal protein VIM through deubiquitylation. This mechanistic insight not only advances our understanding of LUAD metastasis but also opens avenues for targeted interventions aimed at disrupting this specific molecular axis.

There are many pathways that regulate the progression and metastasis of LUAD, such as TGF-β^37^ or Wnt signaling^38^. Comparing the c-Myc-XRCC2-VIM axis to other known pathways involved in LUAD progression and metastasis is crucial for contextualizing its significance. However, its elucidation as a direct transcriptional cascade linking c-Myc to VIM regulation through XRCC2 represents a distinct mechanistic pathway involved in LUAD metastasis. Moreover, combinatorial inhibition of c-Myc and Dox reduced metastasis in vivo, supporting clinical potential.

Despite the promising findings, certain limitations should be acknowledged. The reliance on preclinical models for inhibitor studies underscores the need for further validation in patient-derived models and clinical cohorts. Additionally, expanding the validation of the HR scoring system in independent cohorts will strengthen its reliability and generalizability.

Overall, our study advances our understanding of HRGs in LUAD, offering novel insights into their prognostic and therapeutic implications. The identified biomarker XRCC2, along with the proposed c-Myc-XRCC2-VIM axis, holds significant promise for guiding clinical decision-making and improving outcomes for LUAD patients. Further investigations into the regulatory networks and clinical applications of these findings are warranted for their successful integration into clinical practice.

## Methods

### Datasets acquisition and pre-processing

We utilized the “GDCquery” procedure of TCGAbiolinks package^39^ to obtain the expression profiles of pan-cancer in TCGA portal, including TCGA-LUAD patients. Similarly, the clinical features, such as age, overall survival (OS), and OS time, were also downloaded by “GDCprepare_clinic” procedure. The simple nucleotide variations (SNV) data of TCGA-LUAD cohort were acquired from the GDC portal (https://portal.gdc.cancer.gov/). The maftools package^40^ was utilized to process SNV data and draw waterfall plots of mutated genes. Three external lung cancer cohort (GSE13213, GSE31210, and GSE68465) were collected through the Gene Expression Omnibus (GEO) database (https://www.ncbi.nlm.nih.gov/geo/). Clinical characteristics of the LUAD cohorts, encompassing patient counts, clinical stage, treatment, and other clinical parameters, were comprehensively detailed in Supplementary Table 1. A diagram illustrating the different patient cohorts and their respective analyses is shown in Supplementary Fig. 1.

The 19 key HRGs were obtained based on previous literature^3,13^. For instance, BRCA2, a well-known breast cancer susceptibility gene, plays a crucial role in DNA repair processes. It is recruited to DNA double-strand breaks (DSBs) through interactions with BRCA1, BRCA1-associated RING domain protein 1 (BARD1), and partner and localizer of BRCA2 (PALB2), facilitating the loading of the recombinase RAD51 onto single-stranded DNA (ssDNA). Furthermore, the complex consisting of RAD51B, RAD51C, RAD51D, and XRCC2 forms the BCDX2 complex, which is integral to the homologous recombination repair mechanism.

### Enrichment analysis and survival analysis

Gene set enrichment analysis (GSEA)^41^ was employed to compare the differences between the 524 tumor tissues and 48 normal tissues from the TCGA-LUAD cohort, focusing on three HR-related gene sets (DNA recombination, homologous recombination, recombinational repair) retrieved from the MSigDB database (downloaded on November 5, 2023). The “surv_cutpoint” function of the survminer package was conducted to determine the optimal cut-off value of each HRG for survival analysis. Kaplan-Meier (KM) product limit method was employed to compare OS probabilities of the distinct subgroups and log-rank test was used to determine the *p*-value. A functionally grouped network of the HRGs was constructed based on gene ontology (GO) terms using the ClueGO plugin^42^ for Cytoscape software.

### Identification of HR-related subtypes in LUAD

Based on expression levels of 19 HRGs, we identified two HR-related subtypes in TCGA-LUAD cohort using the ConsensusClusterPlus package^43^ with the parameters of clusterAlg = “hc”, distance = “spearman” and reps = “500”. The principal component analysis (PCA) algorithm was conducted to visualize the distribution of the two LUAD subtypes. We used the limma package^44^ to obtain the differentially expressed genes (DEGs) and the clusterProfiler package^45^ to explore the biological processes of DEGs.

### HR scoring system

To provide better personalized guidance for individual prognosis, we developed a HR scoring system. Initially, we performed univariate Cox regression analysis on the TCGA-LUAD cohort to identify genes significantly associated with clinical outcomes. Based on the principle of *p*-value < 0.05, we obtained 5 candidate genes (XRCC2, RAD51, BRCA1, FANCA, and CHEK1). The hazard ratios (HR), 95% confidence intervals (CI) and *p*-value of these 5 genes were shown in Supplementary Table 2. Then, we conducted the gene set variation analysis (GSVA) to calculate HR scores for each patient in TCGA-LUAD and other cancer types. The patients were dichotomized into high and low HR scores groups based on the median. We compared HR scores between tumor and normal tissue samples across multiple cancer types using data from the GSCA database^46^.

### LUAD tissue samples

From 2015 to 2019, paired LUAD tissues and adjacent non-cancerous lung tissue samples (96 pairs) were collected from patients at Hunan Cancer Hospital with informed consent. The patients were diagnosed with LUAD based on their histological and pathological characteristics. The experiments were carried out according to the ethical guidelines of the Helsinki Declaration. Ethical permissions were granted by the institutional review board of Hunan Cancer Hospital of Central South University. All patients provided their informed written consent to participate in this study.

### Immunohistochemistry (IHC)

For IHC analysis, we used paraffin-embedded tissue sections derived from these clinical samples, with detailed patient information provided in Supplementary Table 3. These sections underwent a baking process at 60 °C for 6 h, followed by dewaxing and rehydration using xylene and a gradient of alcohol concentrations (absolute ethanol I, absolute ethanol II, 95% ethanol, 85% ethanol, and 75% ethanol). Subsequently, antigen retrieval was performed by boiling the prepared antigen retrieval solution in a beaker, and the slides were immersed and boiled for 20 min. Following cooling, endogenous peroxidase activity was quenched with a 3% hydrogen peroxide solution. The tissue sections were then subjected to an overnight incubation at 4 °C with a primary antibody against XRCC2 (ImmunoWay, YT4918, dilute at 1:100). Protein localization and staining intensity were visualized using diaminobenzidine and scored by pathologists.

### Quantitative real-time PCR (RT-qPCR)

Total RNA extraction from cultured cancer cells utilized TRIzol, following standard protocols. Subsequently, cDNA synthesis employed the SuperScript III First Strand cDNA Synthesis System, utilizing 500 ng of total RNA. Quantitative PCR was conducted using the TransStart Green qPCR SuperMix (China) on the qPCR system, following the manufacturer’s instructions.

The primer sequences for the target genes are as follows:

c-Myc:

Forward: GGGAGTTGGGAGGAAGGTGAGG

Reverse: TGGTTGTGAAGGCAGCAGAAGC

XRCC2:

Forward: TGCTTTATCACCTAACAGCACG

Reverse: TGCTCAAGAATTGTAACTAGCCG

### Co-immunoprecipitation (Co-IP) assays

Following expansion of a substantial number of A549 lung cancer cells, proteins were collected and extracted. Magnetic beads were added to the protein solution and incubated at 4 °C for 30 min. Subsequently, the magnetic beads were separated using a magnetic rack, and the remaining protein solution was transferred to a new EP tube. The protein solutions were divided into two groups: the IgG group and the target molecular antibody group. Corresponding antibodies (XRCC2: Santa Cruz Biotechnology, sc-365854, 1:1000. Vimentin: Wanlelbio, WL01960, 1:1000) were added to each group and incubated overnight on a shaking table at 4 °C. A suitable amount of magnetic bead solution was divided into two groups, washed twice with pre-cooled PBS, and added to the protein-antibody complex, reacting at 4 °C for approximately 4–6 h. The EP tube was placed on a magnetic rack to separate the magnetic beads from the protein solution. After separation, the solution underwent boiling at 100 °C for 10 min until protein denaturation. Following centrifugation and magnetic bead separation, the upper liquid was collected for subsequent Western blot detection. All blots and gels were derived from the same experiment and were processed in parallel. Images of uncropped and unprocessed scans of the immunoblots are included in Supplementary Figs. 2–5.

### Silver nitrate staining

In this experiment, the Beyotime rapid silver dyeing kit (P0017S) was employed for silver staining of the gel obtained through electrophoresis. The subsequent differential bands were acquired for mass spectrometry analysis and detection. The procedure involved the following steps:

The gel blocks post-electrophoresis were placed in a clean box and fixed at room temperature on a shaking table for 2 h. Subsequently, after absorbing the fixed solution and cleaning with ethanol, the gel blocks were rinsed twice with ddH2O. Following water absorption, silver sensitizing solution was added for 2 min. After absorbing and discarding ddH2O, an appropriate volume of silver solution was added, and incubation ensued at room temperature for 10 min. The previous liquid was discarded, and the gel blocks were washed twice. The reaction was terminated after absorbing and discarding water, by adding silver dye coloring solution and incubating for 3–10 min until clear and distinct protein bands emerged. Finally, the differentiated bands were photographed in a well-lit environment and excised for subsequent mass spectrometry analysis.

### Immunofluorescence

Cells were fixed on a cell pad using 4% paraformaldehyde for 15–30 min, cleaned with PBS to remove excess fixative. A 0.1–0.5% Triton X-100 PBS solution enhanced antibody penetration. The sample was blocked with a PBS solution containing bovine serum albumin or other blockers for 30 min to 1 h. Overnight incubation at 4 °C with diluted primary antibodies was followed by PBS cleaning to remove unbound antibodies. Fluorescently labeled secondary antibodies were added and incubated for 1-2 h, with PBS cleaning to remove unbound secondary antibodies. Nuclei were stained with DAPI or other dyes. After covering with anti-fading sealant, fluorescence microscopy was used for observation and image capture to analyze fluorescence signal distribution and intensity.

### Scratch test

After applying the protein sample, electrophoresis was run at 60 V for 30 min, and then switched to 100 V until the marker reached the plate bottom. A PVDF membrane, which had been activated in methanol, was used to transfer the proteins at 100 V for 90 min. Following the transfer, the membrane was sealed with 5% skim milk for 1-2 h, cleaned with PBST, and incubated with the primary antibody overnight at 4 °C. After removing the primary antibody, the secondary antibody was applied for 1-2 h at room temperature. Finally, post-secondary antibody removal and luminous development were carried out.

### Transwell invasion experiment

When the confluency of cells reached 80–90%, the cells were harvested by trypsin digestion. After resuspending them in complete medium and centrifuging at 1000 rpm for 5 min, the supernatant was discarded, the cells were resuspended, and their count was determined. 2 × 10^5^ cells were seeded into the upper chamber of a Transwell apparatus, and medium containing 15% FBS (with double antibiotics) was added to the lower chamber. The cells were incubated for 2 days in a 37 °C, 5% CO2 incubator. A clean cotton swab was used to remove the non-migrated cells from the inner chamber of the upper well. The cells were fixed for 15 min, then stained with 0.1% crystal violet solution for 15 min at room temperature, followed by washing with DPBS. The cells were observed and counted under an optical microscope, and statistical analysis was performed.

### Construction of transplanted tumor in nude mice

We established an A549 cell line with stable XRCC2 gene knockout via shRNA transfection, which was then expanded in vitro. For the transplantation, cells in exponential growth phase were harvested, washed with DPBS, and enzymatically dissociated using pancreatin for 1–3 min. The reaction was halted using DMEM medium with 5% fetal bovine serum, and cells were pelleted by centrifugation. The pellet was resuspended in serum-free DMEM or PBS, adjusting the concentration to 2 × 10^6^ cells per injection. The prepared A549 cell suspension was administered intravenously into the tail vein of nude mice using a 1 mL insulin syringe (100 μL/mouse). For the metastatic tumor model, mice were euthanized 20 days post-transplantation to harvest lungs, liver, and spleen for subsequent analyses.

In drug treatment studies, administration commenced on day three post-cell transplantation, with dosages set at 10 mg/kg for Doxorubicin (Dox) and 20 mg/kg for 10058-F4. Mouse weight and health status were monitored every 3 days, with regular changes to feed and bedding. After 24 days from transplantation, mice were euthanized via cervical dislocation, and organs were extracted for photographic documentation and further experiments.

We confirm that this study complies with all relevant ethical regulations on animal experimentation and research. Animal experiments were performed according to protocols approved by the Animal Ethics Committee of Hunan Cancer Hospital.

### Statistical analysis

All statistical analyses utilized R software or GraphPad Prism 8. Student’s *t* test determined statistical differences between the experimental and control groups. One-way ANOVA, followed by Tukey’s post hoc test, assessed differences among multiple groups. Unless otherwise stated, significance was defined as *P* < 0.05.

### Supplementary information

Supplementary Information

## Data Availability

This study did not generate any new data. All the data used in this study were sourced from the public databases. All relevant datasets used in this study are summarized in Supplementary Fig. 1.

## Code availability

This study employed publicly accessible packages in R version 4.2.1. The code for data analysis and figure creation featured in this publication is available upon request from the corresponding authors.
