# Supplementary material for: XRCC2 driven homologous recombination subtypes and therapeutic targeting in lung adenocarcinoma metastasis
Source: NPJ Precis Oncol. 2024 Aug 1;8:169. doi: 10.1038/s41698-024-00658-y (PMC11294482; doi:10.1038/s41698-024-00658-y)

Supplementary Table 1 Clinical features of patients

Clinical features of patients in TCGA-LUAD (n = 515)

**Vital status**	
Alive	330 (64.1)
Dead	185 (35.9)
**Age**	
< 65	221 (44.6)
>= 65	275 (55.4)
**Gender**	
Female	277 (53.8)
Male	238 (46.2)
**Stage**	
Stage I	277 (54.5)
Stage II	121 (23.8)
Stage III	84 (16.5)
Stage IV	26 (5.1)
**T**	
T1	169 (33.0)
T2	279 (54.5)
T3	45 (8.8)
T4	19 (3.7)
**N**	
N0	331 (65.8)
N1	96 (19.1)
N2	74 (14.7)
N3	2 (0.4)
**M**	
M0	347 (93.3)
M1	25 (6.7)
**New tumor event**	
No	277 (66.0)
Yes	143 (34.0)
**Radiation therapy**	
No	372 (86.5)
Yes	58 (13.5)

Clinical features of patients in GSE13213 (n = 117)

**Vital status**	
Alive	68 (58.1)
Dead	49 (41.9)

**Age**	
< 65	76 (65.0)
>= 65	41 (35.0)
**Gender**	
Female	57 (48.7)
Male	60 (51.3)
**Stage**	
Stage I	79 (67.5)
Stage II	13 (11.1)
Stage III	25 (21.4)
**EGFR Status**	
Mut	45 (38.5)
Wt	72 (61.5)
**K-ras Status**	
Mut	15 (12.8)
Wt	102 (87.2)
**P53 Status**	
Mut	38 (32.8)
Wt	78 (67.2)
**Relapse**	
No	58 (50.0)
Yes	58 (50.0)

Clinical features of patients in GSE31210 (n = 226)

**Vital status**	
alive	191 (84.5)
dead	35 (15.5)
**Age**	
< 65	164 (72.6)
>= 65	62 (27.4)
**Gender**	
female	121 (53.5)
male	105 (46.5)
**Stage**	
I	168 (74.3)
II	58 (25.7)
**Smoking status**	
Ever-smoker	111 (49.1)
Never-smoker	115 (50.9)
**Relapse**	

not relapsed	162 (71.7)
relapsed	64 (28.3)

Clinical features of patients in GSE68465 (n = 443)

**Vital status**	
Alive	207 (46.7)
Dead	236 (53.3)
**Age**	
< 65	214 (48.3)
>= 65	229 (51.7)
**Gender**	
Female	220 (49.7)
Male	223 (50.3)
**T**	
T1	150 (34.0)
T2	251 (56.9)
T3	28 (6.3)
T4	12 (2.7)
**N**	
N0	299 (68.0)
N1	88 (20.0)
N2	53 (12.0)
**Smoking history**	
Currently smoking	32 (9.2)
Never smoked	49 (14.0)
Smoked in the past	268 (76.8)
**Progression or relapse**	
No	157 (43.4)
Yes	205 (56.6)
**Adjuvant chemotherapy**	
No	341 (79.3)
Yes	89 (20.7)
**Adjuvant radiotherapy**	
No	364 (84.8)
Yes	65 (15.2)

Supplementary Table 2 Univariate Cox regression results

genes	HR	pvalue	HR (95% CI)
RAD51	1.7946	0.000516	1.7946 (1.2901, 2.4965)
CHEK1	1.7524	0.000546	1.7524 (1.275, 2.4087)
BRCA1	1.4781	0.027647	1.4781 (1.0439, 2.0928)
XRCC2	1.3185	0.035041	1.3185 (1.0196, 1.7051)
FANCA	1.4144	0.049454	1.4144 (1.0008, 1.999)

Supplementary Table 3 Clinical features of LUAD patients in IHC analysis (n = 96)

**5-year survival**	
No	30 (31.2)
Yes	66 (68.8)
**Age**	
< 65	43 (44.8)
>= 65	53 (55.2)
**Gender**	
Female	45 (46.9)
Male	51 (53.1)
**T**	
T1	69 (71.9)
T2	16 (16.7)
T3	6 (6.2)
T4	5 (5.2)
**N**	
N0	76 (79.2)
N1	15 (15.6)
N2	5 (5.2)
**M**	
M0	89 (92.7)
M1	7 (7.3)
**Stage**	
Stage I	69 (71.9)
Stage II	18 (18.8)
Stage III	5 (5.2)

Stage IV	4 (4.2)
**Smoking status**	
No	45 (46.9)
Yes	51 (53.1)

Supplementary Figure 1 A diagram showing what patients are used for what analysis.

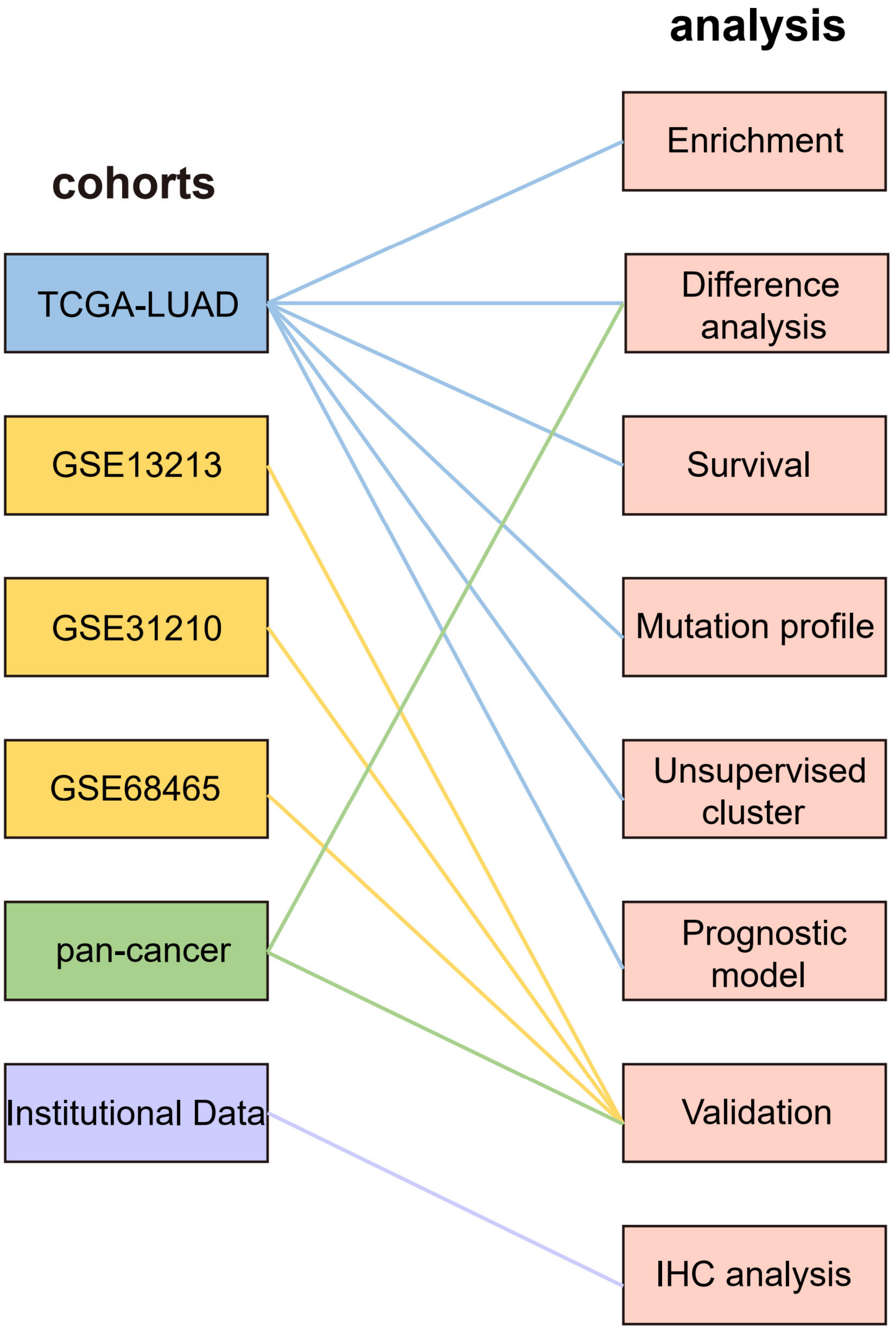

Supplementary Figure 2 Full-sized scans of immunoblots in Figure 6a-f.

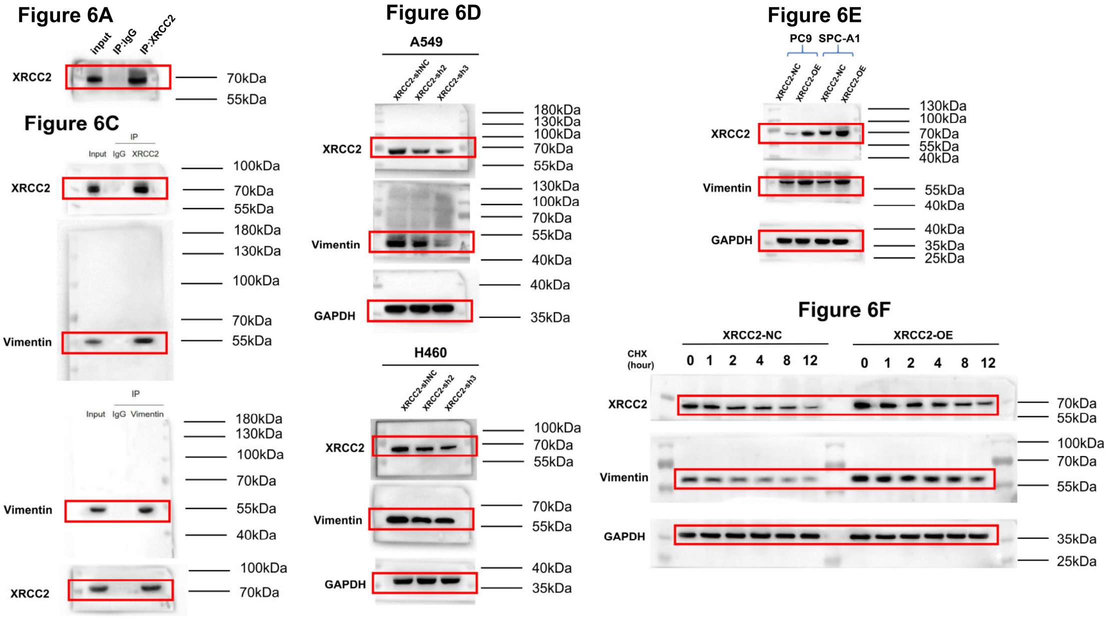

Supplementary Figure 3 Full-sized scans of immunoblots in Figure 6g-h.

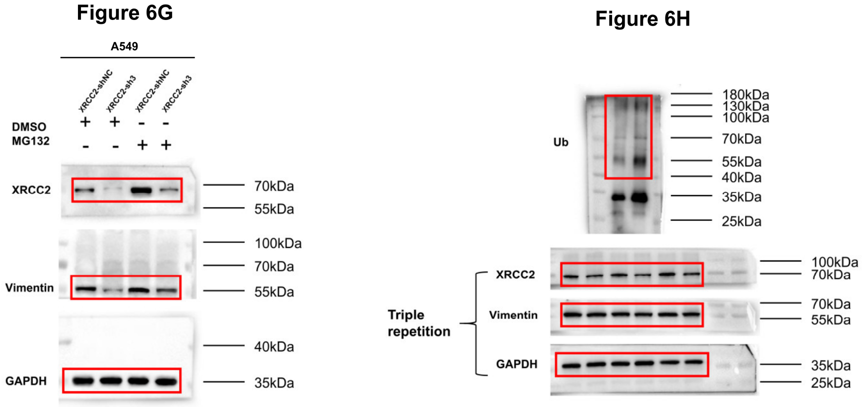

Supplementary Figure 4 Full-sized scans of immunoblots in Figure 8f.

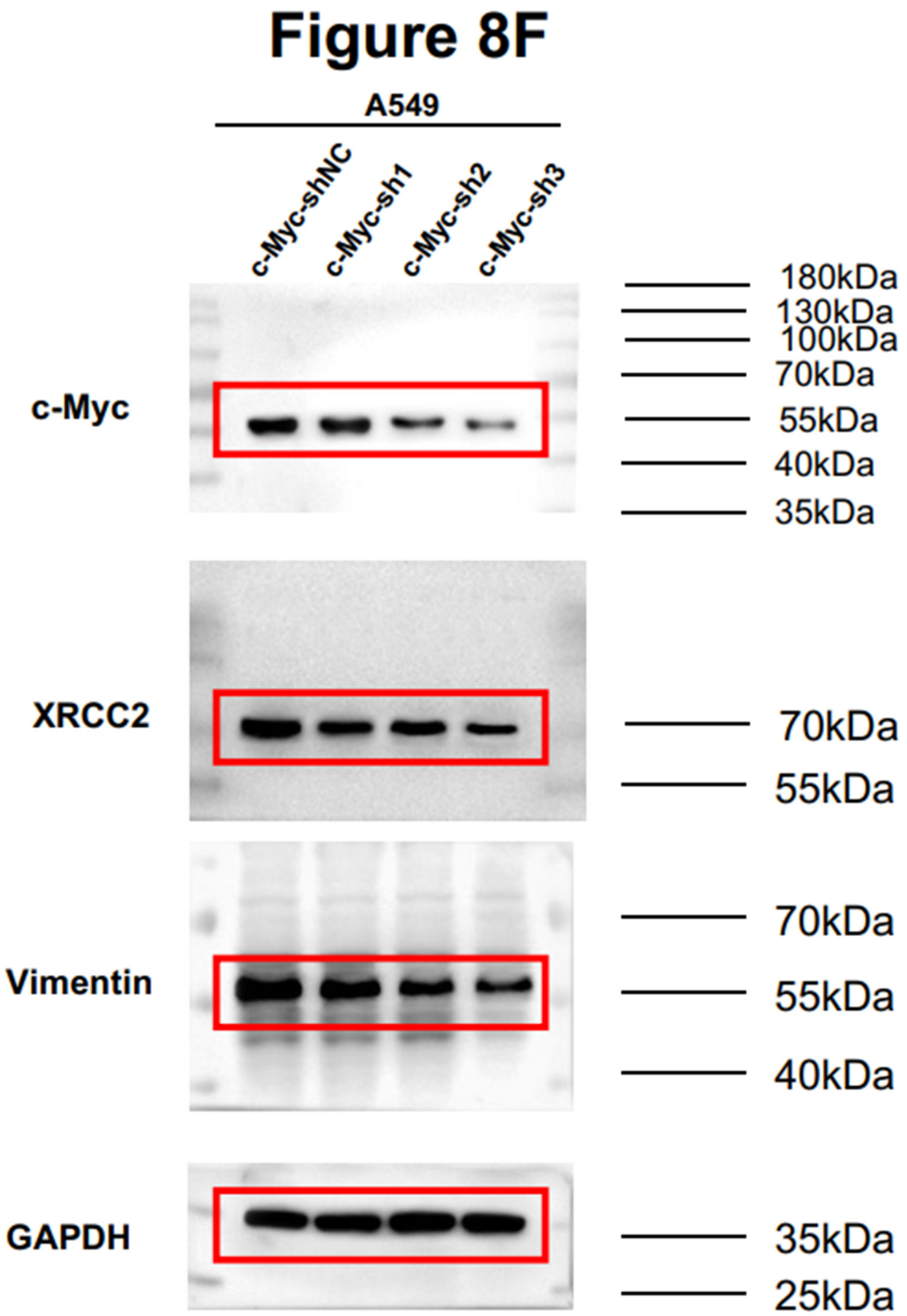

Supplementary Figure 5 Full-sized scans of immunoblots in Figure 9g-h.

Figure 9G

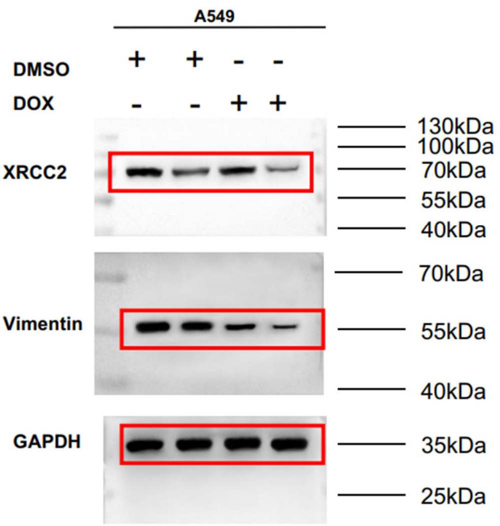

Figure 9H

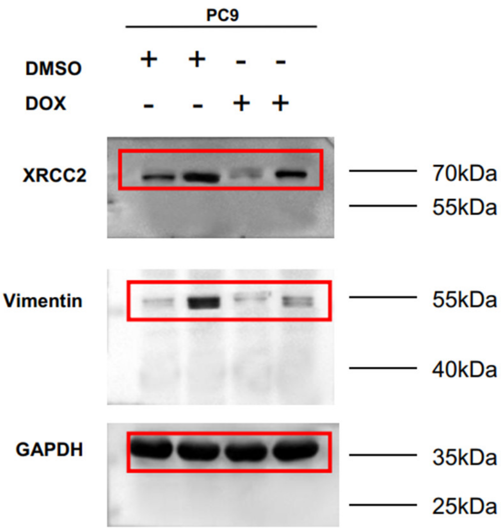

Supplement: Supplementary file 1 — Supplementary Information [file 41698_2024_658_MOESM1_ESM.pdf]
